# Vitamin D/CD46 Crosstalk in Human T Cells in Multiple Sclerosis

**DOI:** 10.3389/fimmu.2020.598727

**Published:** 2020-11-24

**Authors:** Justin Killick, Joanne Hay, Elena Morandi, Sonja Vermeren, Saniya Kari, Thibault Angles, Anna Williams, Jan Damoiseaux, Anne L. Astier

**Affiliations:** ^1^Centre for Inflammation Research, University of Edinburgh, Edinburgh, United Kingdom; ^2^Edinburgh Centre for MS Research, University of Edinburgh, Edinburgh, United Kingdom; ^3^Centre de Physiopathologie Toulouse-Purpan (CPTP), INSERM U1043, CNRS U5282, Université de Toulouse, Toulouse, France; ^4^Centre for Regenerative Medicine, University of Edinburgh, Edinburgh, United Kingdom; ^5^Central Diagnostic Laboratory, Maastricht University Medical Center, Maastricht, Netherlands

**Keywords:** vitamin D, multiple sclerosis, type I regulatory T cells, adhesion, CD46

## Abstract

Multiple sclerosis (MS) is a chronic inflammatory disease of the central nervous system (CNS), in which T-cell migration into the CNS is key for pathogenesis. Patients with MS exhibit impaired regulatory T cell populations, and both Foxp3+ Tregs and type I regulatory T cells (Tr1) are dysfunctional. MS is a multifactorial disease and vitamin D deficiency is associated with disease. Herein, we examined the impact of 1,25(OH)2D3 on CD4+ T cells coactivated by either CD28 to induce polyclonal activation or by the complement regulator CD46 to promote Tr1 differentiation. Addition of 1,25(OH)2D3 led to a differential expression of adhesion molecules on CD28- and CD46-costimulated T cells isolated from both healthy donors or from patients with MS. 1,25(OH)2D3 favored Tr1 motility though a Vitamin D-CD46 crosstalk highlighted by increased VDR expression as well as increased CYP24A1 and miR-9 in CD46-costimulated T cells. Furthermore, analysis of CD46 expression on T cells from a cohort of patients with MS supplemented by vitamin D showed a negative correlation with the levels of circulating vitamin D. Moreover, t-Distributed Stochastic Neighbor Embedding (t-SNE) analysis allowed the visualization and identification of clusters increased by vitamin D supplementation, but not by placebo, that exhibited similar adhesion phenotype to what was observed *in vitro*. Overall, our data show a crosstalk between vitamin D and CD46 that allows a preferential effect of Vitamin D on Tr1 cells, providing novel key insights into the role of an important modifiable environmental factor in MS.

## Introduction

Vitamin D deficiency is a growing public health problem that is proposed as a contributing factor in several chronic inflammatory diseases. Vitamin D3 (cholecalciferol) is formed in the skin from 7-dehydrocholesterol following exposure to the ultraviolet radiation in sunlight. It is converted by hydroxylation in the liver to 25-hydroxyvitamin D3 (25(OH)D3) (calcidiol). This is followed by a second hydroxylation in the kidney to 1,25-dihydroxyvitamin D3 (1,25(OH)2D3) (calcitriol) which is the active form of vitamin D. Vitamin D metabolism is controlled by several enzymes including the cytochrome P450 monooxygenase 25(OH)D 1α hydroxylase (CYP27B1; 1α(OH)ase), which metabolizes 25(OH)D3 to 1,25(OH)2D3, while CYP24A1 converts it back to an inactive form for further degradation. Importantly, these enzymes are also active in leukocytes enabling them to regulate the presence of 1,25(OH)2D3 in the micro-environment (1). Indeed, 1,25(OH)2D3 has immunomodulatory roles and exerts a direct action on T cells (2). T cell activation induces the expression of the nuclear Vitamin D receptor (VDR), which is required for T cell activation (3). 1,25(OH)2D3 decreases secretion of IFNγ, increases IL-10 production and generates both conventional CD25+Foxp3+ regulatory T cells (Tregs) and IL-10-secreting Type I regulatory cells (Tr1) (4–7), which are essential for immune homeostasis (8, 9).

Multiple sclerosis (MS) is a complex autoimmune inflammatory disease of the central nervous system (CNS) in which genetic predisposition and environmental factors contribute to disease pathogenesis (10, 11). The key role of the immune system in MS pathogenesis has been highlighted by genome-wide association studies (12), and the efficacy of immune-targeting therapeutic strategies, with a central role for T cells due to effector T cells (Teffs) that recognize auto-antigens and reduced numbers or impaired function of regulatory T cells. Both Foxp3+ Tregs and Tr1, such as those induced by ligation of the complement regulator CD46 (13, 14), are dysregulated in MS (15–17). While CD46 costimulation first promotes Th1 differentiation, as IL-2 accumulates, CD46 switches human T cells from a Th1 to a Tr1 phenotype, characterized by moderate IFN-γ and high IL-10 secretion (13, 14). T cell activation induces secretion of C3b, a ligand for CD46, resulting into autocrine activation of the T cells (14). CD46 induction of the Tr1 phenotype is dysregulated in MS and other chronic inflammatory diseases, as CD46-costimulated T cells in these patients secrete less IL-10 but normal levels of IFN-γ (14, 16, 18–21). Patients with relapsing-remitting MS have lower levels of circulating vitamin D than controls (22, 23). Treatment with 1,25(OH)2D3 suppresses the development and progression of experimental autoimmune encephalomyelitis (EAE), a murine model of MS (5, 24). In MS, Vitamin D supplementation is safe and has been variably associated with improvement of disease (25, 26) and with a modulation of T cell responses (27–29). *In vitro*, 1,25(OH)2D3 promotes CD46 shedding and the switch to Tr1 differentiation, suggesting that it acts in part through its effects on CD46 (7). Furthermore, 1,25(OH)2D3 can restore the correct ratio of IL-10:IFNγ produced by CD46-costimulated T cells from patients with MS (7).

Immune cell trafficking across the blood-brain-barrier (BBB) is a multi-step process that involves the sequential interaction of specific cellular adhesion and signaling molecules, such as the integrin VLA-4 (α4β1, CD49d/CD29), LFA-1 (αLβ2, CD11a/CD18) and other cellular adhesion molecules such as CD226, CD166, CD146, CD6, and CD99 (30). Recruitment of T cells into the CNS is indeed crucial for the pathology of MS and molecules controlling T cell migration represent key targets for therapy. However, blocking immune cell trafficking is associated with potentially fatal side-effects including progressive multifocal leukoencephalopathy due to limitation of the CNS immune surveillance (31). Therefore, it is important to determine whether distinct adhesion molecules drive the migration of effector T cells and/or regulatory T cells into the CNS, to preferentially target migration of pathogenic T cells.

In this study, we analyze the role of 1,25(OH)2D3 on polyclonally activated T cells or Tr1 differentiated T cells, in the context of T cell migration in MS. Overall, our data suggest that Vitamin D favors response to CD46-induced Tr1 cells through increased VDR expression and allowing a better response to Vitamin D that promotes Tr1 cells. This represents a key mechanism to promote immuno-modulation in chronic inflammatory diseases, including MS.

## Materials and Methods

### Cell Purification and Activation

Peripheral Blood Mononuclear Cel (PBMCl) were isolated from blood obtained after informed consent of patients in the relapsing-remitting stage (RRMS, see patient description in **Table 1**, ethical approval SR258) or from age and sex-matched healthy donors (ethical approval AMREC 115-HV-013), and samples were obtained after informed consent according to the Declaration of Helsinki. Purified CD4+ T cells (StemCell, Grenoble, France) (**Supplementary Figure S1**) were activated for four days with pre-coated anti-CD3 (OKT3, 5µg/ml)/anti-CD46 (MC120.6, 10 µg/ml) or anti-CD3/anti-CD28 (CD28.2, Biolegend, 5 µg/ml), and 10 U/ml of rhIL-2 (Cambridge Bioscience, Cambridge, UK) (13). At the beginning of the culture, cells were treated with 10^-7^M 1,25(OH)2D3 (Sigma, Gillingham, UK), a dose previously reported to promote IL-10-producing Tr1 cells (6, 32, 33) or the vehicle control, ethanol.

**Table 1:** Multiple Sclerosis (MS) patient characteristics.

Patient	Age	Gender	EDSS	Treatment
MS1	n/a	M	1	None
MS2	43	F	1	Dimethyl fumarate
MS4	25	M	1	Dimethyl fumarate
MS5	49	F	1.5	None
MS6	33	F	1	None
MS7	41	F	2	Beta Interferon
MS8	39	M	2	Dimethyl fumarate
MS10	32	F	1	Beta Interferon
MS11	46	F	0	Dimethyl fumarate
MS12	55	F	2	Dimethyl fumarate
MS13	33	F	1	Dimethyl fumarate
MS14	39	F	0	Dimethyl fumarate
MS15	31	F	0	Dimethyl fumarate
MS16	37	F	1	Dimethyl fumarate
MS17	53	M	2	Dimethyl fumarate
MS18	49	F	2	Dimethyl fumarate
MS19	32	M	1	Dimethyl fumarate
MS20	44	F	2	Dimethyl fumarate
MS21	21	M	n/a	Dimethyl fumarate
MS22	36	M	1	Dimethyl fumarate
MS23	57	F	7	None

### Patients Supplemented by Vitamin D

Frozen PBMCs from RRMS patients from a former study (34) were obtained and subjected to flow cytometry analysis. Briefly, RRMS patients with relapsing-remitting MS received vitamin D3 supplements (4,000 IU/day) or placebo during 16 weeks and flow cytometry analyses were performed before and after supplementation.

### Flow Cytometry

After T cell activation, T cells were stained with directly labeled antibodies (or isotype controls) as follows: CD46 (FITC, clone: MEM-258, Biolegend), CD11a (PE, clone HI111, Biolegend), CD69 (FITC, clone FN50, Biolegend), CD99 (PE, clone 3B2/TA8, Biolegend), CD29 (PE, clone: TS2/16, Biolegend), CD49d (APC, clone: 9F10, Biolegend), CXCR3 (PE human, Miltenyi Biotech), CD226 (APC, clone 11A8, Biolegend), CD146 (APC, clone P1H12, Biolegend), CD166 (PE, clone 3A6, Biolegend), TIGIT (APC, clone: A15 15 36, Biolegend). Cells were analyzed using a FACSCalibur or a 5 laser LST Fortessa (BD Biosciences). A live-dead marker (LIVE/DEAD™ Fixable Far Red Dead Cell Stain, Thermofisher) was added to the cells to exclude dead cells. The delta geometric mean is shown (MFI staining – MFI isotype). Representative staining are shown in **Supplementary Figure S1**. For the cohort of patients supplemented with vitamin D, PBMCs were analyzed with a panel of antibodies consisting of CD3-AF700, CD4-FITC, CD25-BV605, CD127-BV421, CD45RA-BV510, Foxp3-PE, CD46-PE/cy7, CD226-PE-dazzle 594, CD146-APC, CD162-BV711, CD6-BV785. Viable cells were selected using the NIR-ZOMBIE APC-Cy7 (Biolegend) (**Supplementary Figure S1C**). Cells were analyzed using a Fortessa (BD Biosciences) and analysis was done using FlowJo and t-SNE clustering.

### Analysis of Flow Cytometry Data With t-SNE and Phenograph

Data were first analyzed using FlowJo with FlowAI that cleans unwanted events by evaluating three different properties (abrupt changes in the flow rate, instability of signal acquisition and outliers in the lower limit and margin events in the upper limit of the dynamic range) (35). The number of live CD3+CD4+ T was reduced in order to normalize the number of events from each file to the concatenated total. Samples were randomly downsampled to 20,000 events per sample for *ex vivo* PBMCs (10,000 events per sample for activated cells). The concatenated population contained a total of 360,000 cells for ex vivo PBMCs (140,000 per sample for activated cells. A t-Distributed Stochastic Neighbor Embedding (tSNE) was then run on the concatenated file to allow the visualization of complex multi-dimensional data in fewer dimensions. Events with a similar multidimensional expression pattern group together within the dimensionally reduced data space. CD3, CD4 and Viability Dye were removed from the t-SNE and Phenograph analysis. In order to choose the analysis parameters for the t-SNE and Phenograph analysis, the data were run with increasing perplexity (from 10 to 10 starting at 30 as the default value) for t-SNE and increasing k value (from 5 to 5, starting at 30) for Phenograph (36). From a perplexity of 80 and above, the clustering did not significantly change anymore. We therefore chose 100 and 50 for the k-value (reaching a plateau). Opt-SNE, an automated toolkit for t-SNE parameter selection (37) was run with the following parameters: perplexity = 100 (the number of nearest neighbors), iteration = 1000 (maximum number of iterations) and Eta = 44,099 (Learning rate). In each figure, all samples were derived from the same t-SNE run. Phenograph was also run on the concatenated files to determine phenotypic similarities between cells according to k-nearest neighbors (k=50) and automatically identify clusters (38).

### Chemotaxis Assay

The ability of activated CD4^+^ T cells to migrate toward CXCL11 was monitored by a chemotaxis assay using Transwell inserts (6.5 mm insert, 3 μm filter, Costar). 600 μl/well RPMI or CXCL11 (100 ng/ml, Recombinant Human CXCL11 (ITAC), carrier-free, Biolegend) was added to the lower chambers. Activated CD4^+^ T cells were resuspended in fresh medium (1 million/ml) and 100 μl was added to the upper chamber of the Transwell inserts, and incubated at 37°C for 3.5 hrs. To evaluate chemotaxis, cells in the bottom chamber were then analyzed by flow cytometry for 1 min to evaluate the number of cells present. Dunn chamber chemotaxis was performed with CD3/CD46-activated T cells allowed to migrate in a gradient of 0-100nM nM CXCL11. Cells were monitored in a temperature-controlled chamber for 30 min by time-lapse imaging using an inverted RMDIB microscope (Leica) equipped with an Orca camera (Hamamatsu) that was controlled by Micromanager image acquisition software (Fiji). The paths of individual cells were tracked using the manual tracking plug-in into ImageJ and tracks analyzed using the chemotaxis tool plug-in into ImageJ (Ibidi).

### Quantification of mRNA and miRNA by qPCR

Subsequent to T cell activation, the T cells were harvested, lysed in TRIzol reagent (Qiagen) and the total RNA isolated using the miRNeasy mini kit (Qiagen) as directed by the manufacturer. The quantity and purity of the extracted RNA was determined using a Nanodrop spectrophotometer and cDNA generated using the QuantiTect reverse transcription kit (Qiagen). Relative mRNA expression of CYP27B1 (*Forward primer: CCAGACAGCACTCCACTCAG, Reverse primer: ACAGAGTGACCAGCGTATTT*), CYP24A1 (*Forward primer: GATTTTCCGCATGAAGTTGGGT, Reverse primer: CCTTCCACGGTTTGATCTCCA*) and VDR (*Forward primer: GTGGACATCGGCATGATGAAG, Reverse primer: GGTCGTAGGTCTTATGGTGGG*) using a Roche Lightcycler with PowerUp SYBR green mastermix (ThermoFisher). The ribosomal protein RPL13A, (Forward primer: ACCGTCTCAAGGTGTTTGACG, Reverse Primer: GTACTTCCAGCCAACCTCGTG) was used as an endogenous control (1). Expression of miR-9 and RNU-44 was assessed using Taqman miRNA assays (Applied Biosystems). Results were analyzed using the (2^-ΔΔCt^) method with normalization against RPL13a and RNU44 expression for mRNA and miR, respectively. Results are expressed as fold change relative to unstimulated cells.

### Statistics

The groups were analyzed using Graphpad Prism software. All data are represented as mean ± standard error of the mean (SEM), and were analyzed using the Wilcoxon test when assessing paired samples, or ANOVA with appropriate *post hoc* analysis for multiple comparisons. A non-parametric Mann-Whitney test or Kruskall-Wallis test was used to compare healthy T cells and MS T cells. Regression analysis was used to test for correlations between vitamin D concentrations and CD46 expression. P-values were considered significant as follows: *: p<0.05 to 0.01, **: 0.001 to 0.01, ***: 0.0001 to 0.001, and ****: < 0.0001.

## Results

### 1,25(OH)2D3 Differentially Modulates Surface Expression of Adhesion Molecules Depending on the Activation Pathway

**Figure 1:**
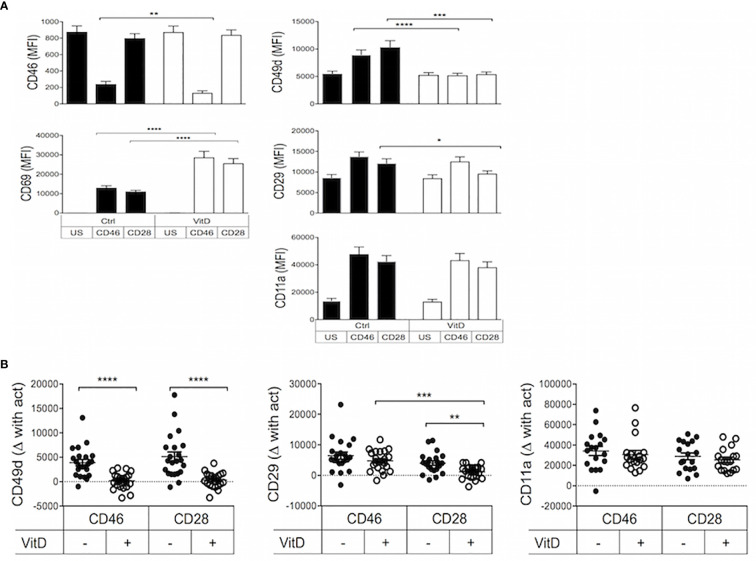
1,25(OH)2D3 differentially modulates integrin surface expression on activated T cells depending on their activation pathway. **(A)** Human CD4+ T cells were left unstimulated (US) or were activated with anti-CD3 and anti-CD28 or anti-CD46 as indicated, in the presence or absence of 1,25(OH)2D3 (VitD). After 4 days, expression of CD46, CD69, CD49d, CD29, and CD11a was assessed by flow cytometry (mean ± SEM, n= 24, one way ANOVA with Holm-Sidak’s multiple comparisons *post hoc* test). **(B)** The change in expression of CD49d, CD29, and CD11a upon activation compared to expression in unstimulated T cells was calculated (mean ± SEM, n= 22, Friedman test with Dunn’s multiple comparisons test). *: p<0.05 to 0.01, **: 0.001 to 0.01, ***: 0.0001 to 0.001, and ****: < 0.0001.

Purified human CD4+ T cells from healthy donors were activated in the presence or absence of 1,25(OH)2D3 by either CD3/CD28 to assess polyclonally activated effector T cells or were costimulated with the complement regulator CD46 to induce Tr1-like cells (13, 14) (**Supplementary Figure S2**). Expression of the integrins CD49d (the α chain of VLA-4, α4), CD29 (the β chain of VLA-4, β1) and CD11a (the α chain of LFA-1, αL), known to be involved in the regulation of T cell trafficking to the CNS, was determined. The expression of CD46 and CD69 was used to assess activation. As previously reported (7), the addition of 1,25(OH)2D3 promoted CD46 shedding upon its ligation (**Figure 1A**) that is required for Tr1 differentiation (39, 40). The two costimulation pathways induced similar expression of the activation marker CD69, and this was strongly increased by the addition of 1,25(OH)2D3. In contrast, 1,25(OH)2D3 inhibited T-cell activation dependent upregulation of CD49d, and significantly decreased CD29 on CD28- but not on CD46-coactivated T cells, and had no significant effect on CD11a expression (**Figure 1A**). To better visualize the modulation of expression, the difference in the levels of CD49d, CD29 and CD11a after activation with CD46 or CD28 costimulation compared to their basal expression in unstimulated T cells is also represented in **Figure 1B**. It was previously reported that 1,25(OH)2D3 increased expression of the skin-homing chemokine receptor CCR10, and decreased expression of the gut-homing trafficking molecules α4β7-integrin and CCR9 (41). In agreement, we also observed that Vitamin D reduced β7-integrin expression on CD28-coactivated T cells (**Supplementary Figure S3A**). In contrast, we found that 1,25(OH)2D3 significantly increased expression of CCR9, but not CCR10, following both costimulation pathways (**Supplementary Figure S3B**), and we did not detect a significantly increased β7-integrin expression after CD46-costimulation as previously reported (42).

**Figure 2:**
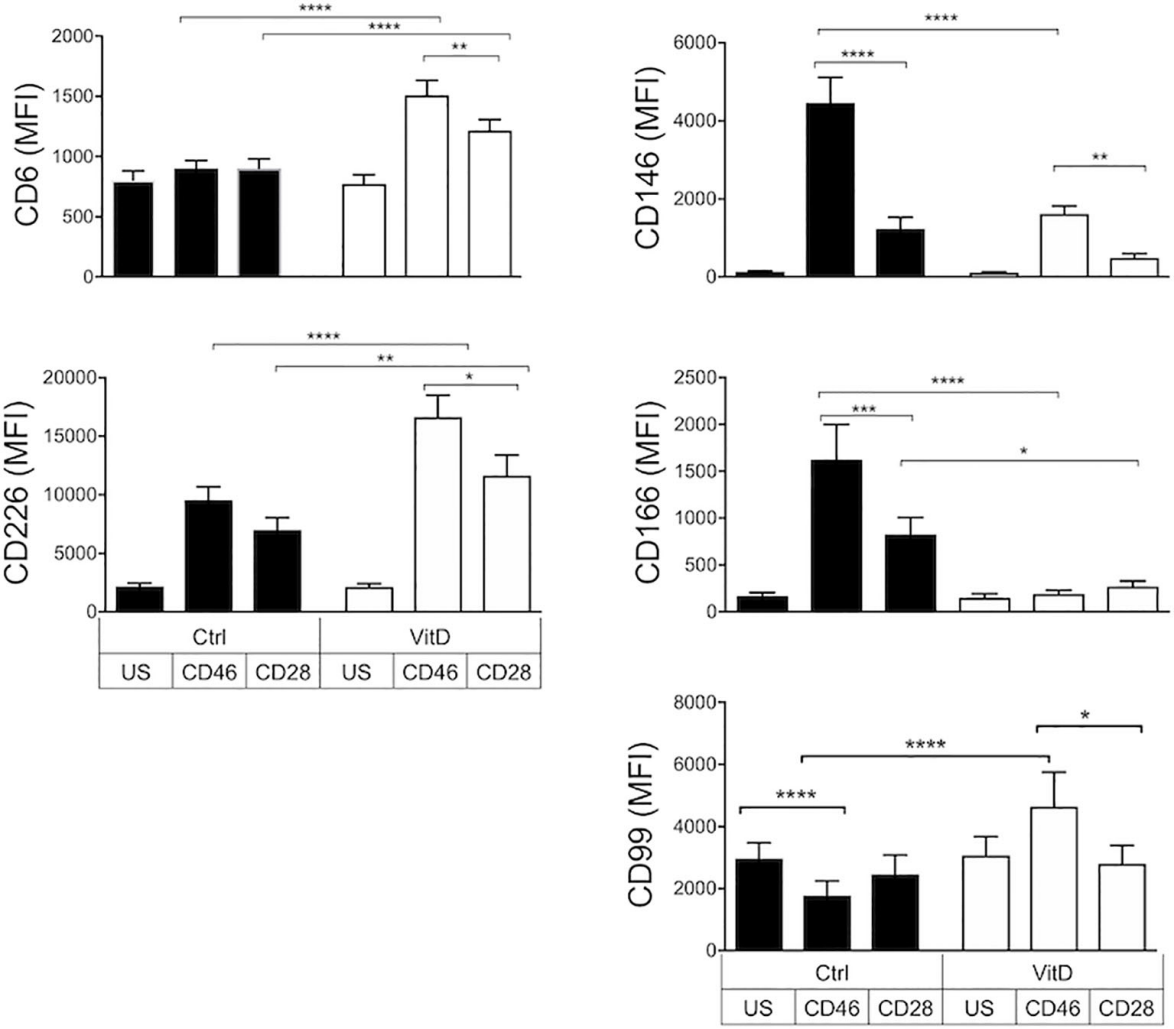
1,25(OH)2D3 differentially modulates the expression of cellular adhesion molecules on activated T cells depending on their activation pathway. Human CD4+ T cells were left unstimulated (US) or were activated with anti-CD3 and anti-CD28 or anti-CD46 as indicated, in the presence or absence of 1,25(OH)2D3 (VitD). After 4 days, expression of CD6, CD226, CD146, CD166, and CD99 was assessed by flow cytometry (mean ± SEM, n= 24 (n= 15 for CD99), one way ANOVA with Holm-Sidak’s multiple comparisons *post hoc* test). *: p<0.05 to 0.01, **: 0.001 to 0.01, ***: 0.0001 to 0.001, and ****: < 0.0001.

We next assessed the effect of 1,25(OH)2D3 on a panel of activation and cellular adhesion molecules that had been previously identified as playing a role in T cell activation and/or migration to the CNS (43–46). First, we observed that, in the absence of 1,25(OH)2D3, CD28 and CD46 costimulations led to a differential modulation of these surface receptors (**Figure 2**). CD46 notably promoted expression of CD226/DNAM-1, CD146/MCAM, and CD166/ALCAM but reduced CD99. Second, 1,25(OH)2D3 had marked effects by increasing CD6, CD226 as well as CD99 expression and decreasing CD146 and CD166 expression. Except for CD166, 1,25(OH)2D3-treated CD46-costimulated T cells expressed higher levels of cellular adhesion molecules compared with CD28-costimulated T cells (**Figure 2**).

**Figure 3:**
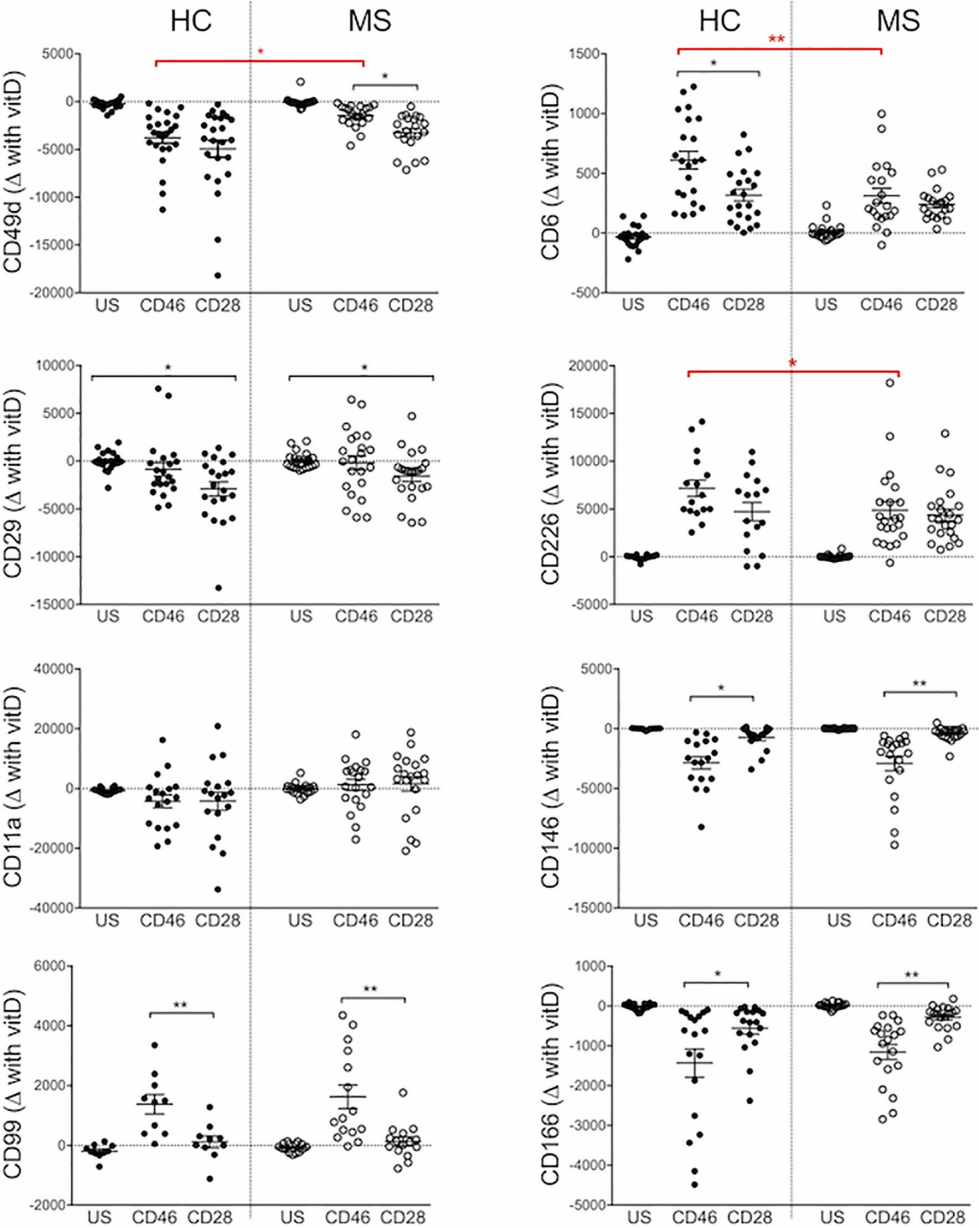
1,25(OH)2D3 exerts similar effects on the phenotype of activated multiple sclerosis (MS) T cells. CD4+ T cells isolated from the cohort of healthy controls (HC) (shown in **Figures 1** and **2**) or patients with relapsing-remitting stage (RRMS) (n=20) were activated with anti-CD3 and anti-CD28 or anti-CD46 as indicated, in the presence or absence of 1,25(OH)2D3 (VitD). The difference in expression of CD49d, CD29, CD11a, CD99, CD6, CD226, CD146, and CD166 induced by 1,25(OH)2D3 is plotted, Kruskall-Wallis test. *: p<0.05 to 0.01 and **: 0.001 to 0.01.

The effect of 1,25(OH)2D3 on T cells isolated from patients with relapsing-remitting MS (RRMS) was next examined (**Supplementary Figure S4**). We calculated the difference in expression of adhesion molecules upon addition of 1,25(OH)2D3 (**Figure 3**). Similar effects of 1,25(OH)2D3 were observed for healthy donors and RRMS patients, although the increase in CD6 and CD226 observed with addition of 1,25(OH)2D3 was reduced in CD46-costimulated MS cells; this also holds for the decrease in CD49d expression. These changes seemed to be independent of disease-modifying treatments (**Supplementary Figure S5**).

Overall, these data demonstrate a distinct modulation of cell adhesion molecules controlling migration by 1,25(OH)2D3 on CD28- and CD46-costimulated T cells. Similar trends were observed on T cells isolated from healthy donors and patients with RRMS, although CD46 costimulation of MS T cells revealed a reduced modulation, likely reflecting the altered CD46 pathway in these patients (16, 18).

### 1,25(OH)2D3 Favors Chemotaxis of CD46-Mediated Tr1 Cells Toward CXCL11 Through a CD46-Vitamin D Crosstalk

**Figure 4:**
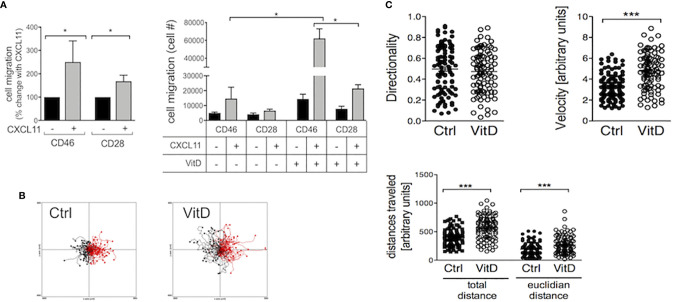
1,25(OH)2D3 favors migration of CD46-activated T cells. **(A)** Human CD4+ T cells were activated as indicated and chemotaxis to CXCL11 assessed by transwells after 3.5 hrs. The left panel shows the increased chemotaxis after normalization to migration in the absence of CXCL11 (n=10, mean ± SEM), and the right panel the numbers of cell migrated when the cells were activated in the presence or absence of 1,25(OH)2D3 (n=6, mean ± SEM). **(B, C)** CD46-costimulated T cells activated in the presence or absence of 1,25(OH)2D3 were added to Dunn chambers with CXCL11 and time-lapsed every 30 s for 30 min. Analysis of the cell movements were performed using the chemotaxis plugin from Ibidi into imageJ. *: p<0.05 to 0.01 and ***: 0.0001 to 0.001.

Chemokine receptors dictate chemotaxis and migration of activated T cells. Notably CXCR3 is implicated in regulating T cell migration in MS and 1,25(OH)2D3 reduces CXCR3 expression in EAE (47). We assessed chemotaxis of activated T cells (upper chambers) toward the CXCR3 ligand CXCL11 (lower chambers). Addition of CXCL11 increased migration of both CD28- and CD46-coactivated T cells (**Figure 4A**, left panel). 1,25(OH)2D3 enhanced CXCL11-mediated chemotaxis, and it favored migration of CD46-costimulated cells compared with CD28-activated T cells (~3 fold increase) (**Figure 4A**, right panel). The effect of 1,25(OH)2D3 on increasing Tr1-like cell motility was confirmed by assessing velocity and distance traveled using Dunn chambers (**Figures 4B, C**).

**Figure 5:**
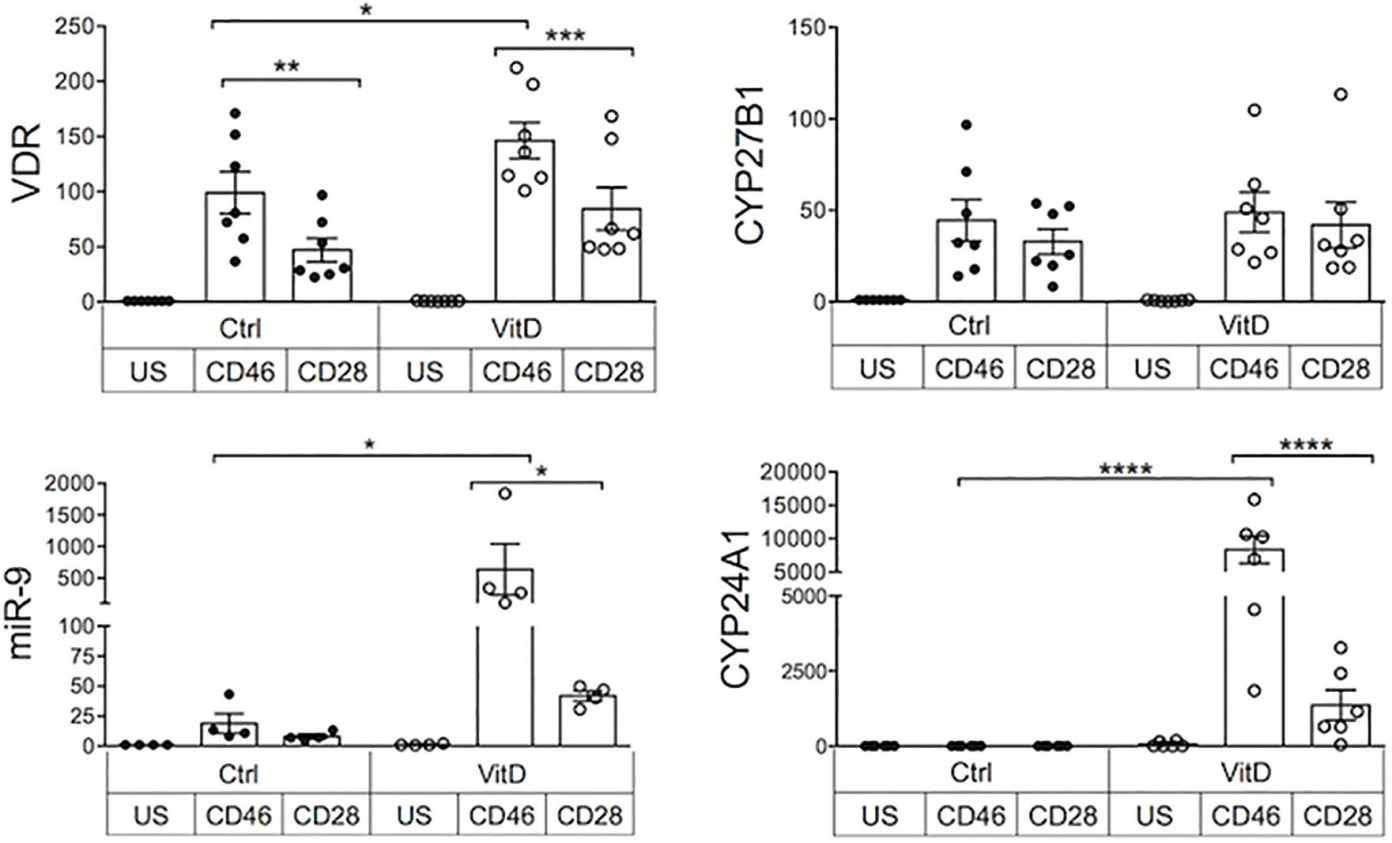
Crosstalk between the CD46 and vitamin D pathways. Human CD4+ T cells were activated as indicated and expression of VDR, miR-9, CYP27B1, and CYP24A1 was quantified by qPCR after 4 days. Relative quantification was calculated according to the expression of the house keeping gene (ΔCt) and the fold change compared to unstimulated samples (2^-ΔΔ^Ct) was calculated. Analysis was performed using one way Anova with Sidak’s multiple comparisons test. *: p<0.05 to 0.01, **: 0.001 to 0.01, ***: 0.0001 to 0.001, and ****: < 0.0001.

The increased motility induced by 1,25(OH)2D3 was not due to increased CXCR3 expression on activated T cells (**Supplementary Figure S6**). The biological functions of 1,25(OH)2D3 are mediated through the VDR. Moreover, the amount of 1,25(OH)2D3 available is regulated by CYP27B1 and CYP24A1. To explain the preferential effect of vitamin D on CD46-costimulated cells compared with CD28-activated cells, the levels of expression of VDR, CYP27B1 and CYP24A1 mRNA in activated T cells were assessed, with and without the addition of 1,25(OH)2D3 (**Figure 5**). We also examined expression of the miRNA miR-9 that regulates VDR expression (48). Differences in their expression were found depending on the costimulatory signals received by the T cells. Increased VDR expression was detected upon T cell activation confirming previous reports (3), but CD46-costimulation doubled VDR expression when compared with CD28-costimulation, and expression was further increased by addition of 1,25(OH)2D3. Expression of miR-9 was increased following CD46-costimulation, and significantly augmented in the presence of 1,25(OH)2D3. CYP27B1 was not significantly increased by CD46 costimulation compared with CD28 and was not modulated by 1,25(OH)2D3, but adding 1,25(OH)2D3 led to a large increase in CYP24A1 in CD46-costimulated T cells, indicating that the increased VDR expression is functional. As 1,25(OH)2D3 promotes the CD46-mediated switch from pro-inflammatory Th1 to regulatory Tr1 (7, 14), these data indicate interplay between the CD46 and Vitamin D pathways, favoring an effect of 1,25(OH)2D3 on Tr1 cells.

### Vitamin D Supplementation Modulates the Expression of Adhesion Molecules on CD4+ T Cells in Patients With MS *In Vivo*

**Figure 6:**
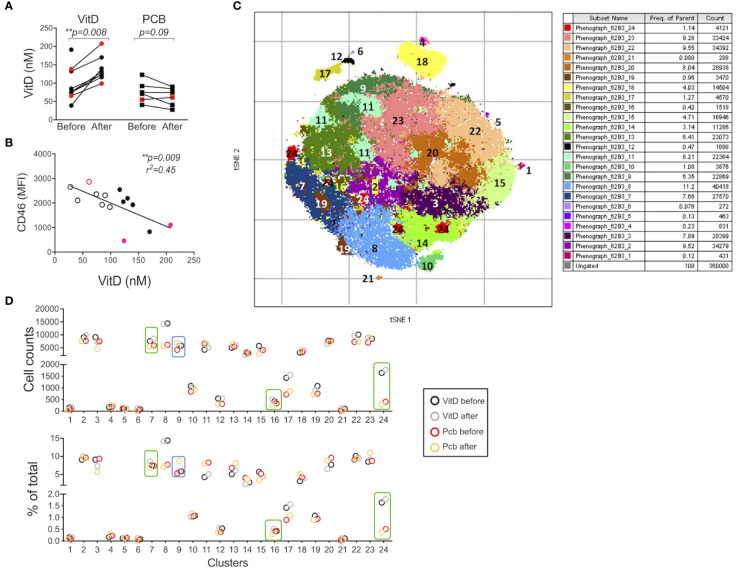
Vitamin D supplementation modulates the phenotype of T cells in patients with multiple sclerosis (MS) *in vivo*. **(A)** 25(OH)D levels in serum before and after a 16-week treatment with vitamin D or placebo (PCB) in a cohort of patients with MS (11 IFNβ-treated, 3 untreated, red dots). **(B)** Correlation between the expression of CD46 on circulating CD3+CD4+ T cells and vitamin D concentration. Placebo: open circles, vitamin D: closed symbols, untreated patients depicted as red dots. **(C)** t-SNE was run on the concatenate file of 360,000 cells (perplexity = 100, iteration = 1000, Eta= 44,099). Phenograph was also run to identify clusters (K=50) in the CD3+CD4+ T cells from five patients supplemented with vitamin D and four placebo (all IFNβ treated). **(D)** Cell counts and percentage of the 24 clusters identified by PhenoGraph (in **C**) in the vitamin D and placebo groups before and after treatment. Boxes identified the clusters for which a differential modulation was observed with vitamin D and placebo.

**Figure 7:**
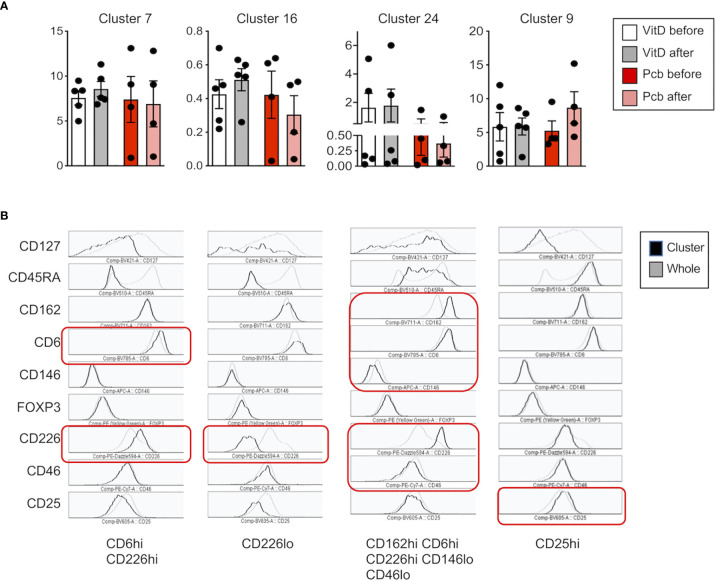
Vitamin D supplementation modulates the expression of adhesion molecules on CD4+ T cells in patients with multiple sclerosis (MS). **(A)** Percentage and **(B)** phenotype of the clusters increased after vitamin D supplementation (clusters 7 and 16), decreased by placebo (clusters 16 and 24) or increased by placebo but stable with vitamin D (cluster 9). The phenotype represented is from the Vitamin D after group and is representative of the four different groups as they represent the phenotype of the cells clustered together.

In order to assess whether Vitamin D was able to modulate expression of adhesion molecules in humans *in vivo*, we next analyzed samples from a cohort of patients with RRMS supplemented by vitamin D3 (or placebo) previously reported (34). Within PBMCs, CD3+CD4+ T cells were analyzed for CD46, CD226, CD6, CD146, CD162/PSGL1, as well as Treg markers (Foxp3, CD25, CD127, CD45RA). Significant increases in circulating vitamin D levels were observed after supplementation (**Figure 6A**). We first analyzed the correlation between the concentration of circulating vitamin D and CD46 expression at the surface of T cells. A significant negative correlation was observed for CD46 expression on CD4+ T cells (**Figure 6B**). Similar trends were observed when the patients were segregated according to vitamin D supplementation (placebo with open circles, vitamin D with closed symbols, untreated patients depicted as red dots). Expression of the adhesion molecules was determined using unsupervised t-SNE analysis and phenograph clustering that allowed the identification of 24 clusters within the CD3+CD4+ population (**Figures 6C, D**, **Supplementary Figure S7**). We selected clusters for which a modulation of the proportion/cell counts was differentially observed after vitamin D treatment or placebo. The clusters 7, 16, and 24 were increased with VitD and/or decreased with placebo (**Figure 7A**). Among these, the cluster 24 reproduced the effects observed *in vitro* when activated by CD46, with reduced CD46 expression associated with an increased expression of CD6 and CD226, and a decreased expression of CD146 (**Figure 7B**). Interestingly, a strong increase in CD162 was also detected in this cluster. We confirmed the regulation of CD162 *in vitro* by CD46 stimulation in the presence of vitamin D (**Supplementary Figure S8**). Reduced CD25 expression was detected on the clusters 7 and 16. In contrast, the cluster 9, which is increased in the placebo group but stable with vitamin D, expressed increased CD25. While an increase of CD6 was observed for the clusters 7, 16, and 24, the upregulation of CD226 was only observed in the clusters 24 and 7, while cells represented in the cluster 16 exhibited a strong downregulation of CD226 expression. This suggests that vitamin D supplementation exerts different effects on CD226 depending on the T cell subsets. Overall, the data show that vitamin D supplementation affects CD46 expression and modulates the adhesion molecules on circulating T cells, with some T cell clusters resembling the phenotype observed after CD46 costimulation *in vitro*. The analysis also allowed the identification of 2 clusters (4 and 18) that corresponded to Tregs (Foxp3+CD25+CD127lo) (**Supplementary Figure S9**). Interestingly, the cluster 4 expressed very high levels of CD146 and increased CD46 expression. However, no significant differences were observed between the placebo and vitamin D supplemented groups as similar increased proportions and cell numbers were detected for both groups (**Supplementary Figure S9**).

### Vitamin D Supplementation Modulates the Profile of Adhesion Molecules Upon In Vitro T Cell Activation

**Figure 8:**
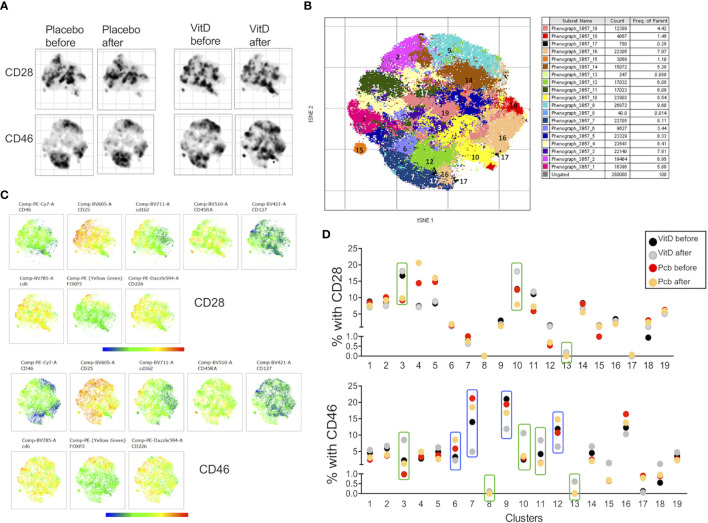
Vitamin D supplementation modulates the phenotype of T cells in patients with multiple sclerosis (MS) after *in vitro* activation. **(A)** t-SNE representation and **(B)** intensity of staining of CD46, CD25, CD162, CD45RA, CD127, CD6, Foxp3, and CD226 in CD3+CD4+ T cell clusters after CD28 or CD46 costimulation of PMBCs from patients with MS supplemented by vitamin D or placebo (n=3 and n=4, respectively). **(C)** visualization of clusters identified by Phenograph on t-SNE-map analysis of the CD3+CD4+ T cells. **(D)** Percentage of the 19 clusters identified by PhenoGraph in the vitamin D and placebo groups before and after treatment. Clusters increased (green boxes) or decreased (blue boxes) after vitamin D supplementation are indicated.

**Figure 9:**
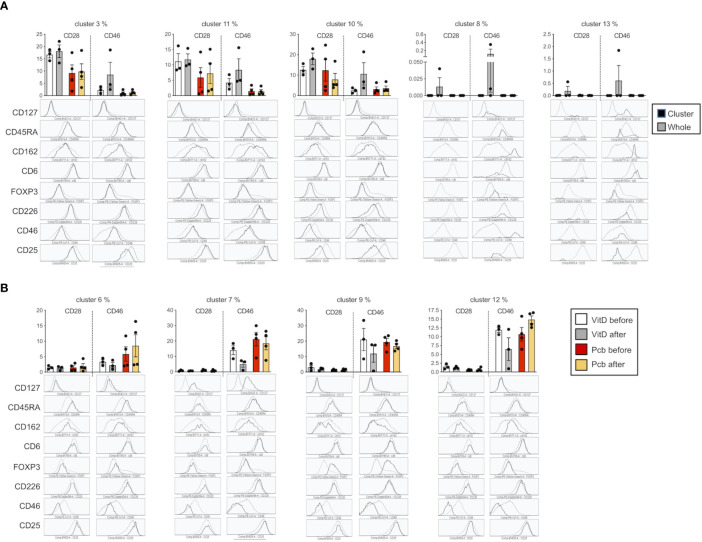
T cell phenotypes modulated by vitamin D in patients with multiple sclerosis (MS) after *in vitro* activation. Percentage and phenotype of the clusters observed after CD28 or CD46 costimulation that are increased **(A)** or decreased **(B)** after vitamin D supplementation but not by placebo. The phenotype represented is from the Vitamin D after group and is representative of the 4 different groups.

We next determined whether vitamin D supplementation could modulate the profile of cell adhesion molecules after T cell activation. We compared the phenotype of the CD3+CD4+ T cells after costimulating the PBMCs *in vitro* with CD28 or CD46. The t-SNE analysis showed, as expected, different clustering depending on the costimulation pathway (**Figures 8A, B**). Interestingly, changes in the clustering were mainly observed after vitamin D supplementation upon CD46 costimulation with minimal changes observed after CD28 coactivation. The phenograph analysis identified 19 clusters, with different proportions depending on the costimulation pathways (**Figure 8C**, **Supplementary Figure 10**). We highlighted the clusters for which an increased or decreased proportion was detected after vitamin D supplementation and not placebo (**Figure 8D**). Analysis of their phenotype shows that most of the clusters that were increased by vitamin D supplementation had lower CD25 expression and increased CD162 levels, with variable levels of CD46, CD226, and CD6 (**Figure 9A**). In contrast, the clusters decreased by vitamin D supplementation exhibited increased CD25 expression and lower CD46 expression with minimal changes in the adhesion molecules (**Figure 9B**). These data show that vitamin D supplementation also affects the profile of T cells upon their reactivation.

## Discussion

**Figure 10:**
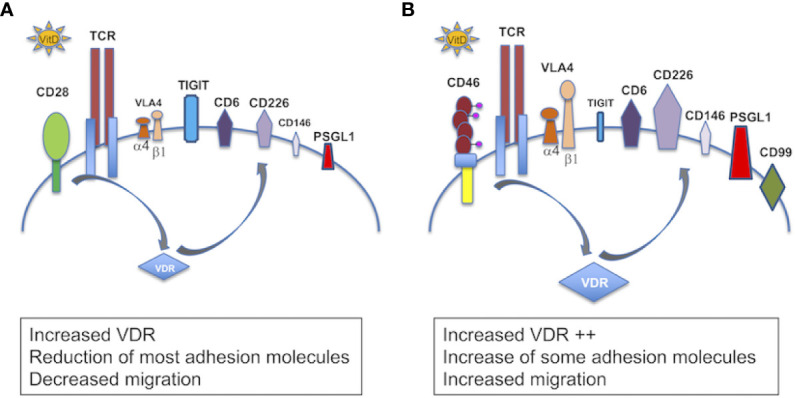
Schema summarizing the role of vitamin D in the modulation of adhesion of T cells. Schema summarizing the effect of 1,25(OH)2D3 on the modulation of expression of adhesion molecules in **(A)** CD28- and **(B)** CD46-costimulated T cells. In the presence of 1,25(OH)2D3, CD28 costimulation increases VDR, decreases expression of VLA4 (CD49d and CD29) and CD146, and moderately induces CD6, CD226 and PSGL1, and promotes TIGIT expression. CD46 costimulation leads to a larger VDR expression, decreases CD49d but maintains CD29, strongly promotes expression of CD6, CD226, PSGL1, and induces CD99. CD46 costimulation also decreases expression of CD146 and represses TIGIT.

In this study, we highlight a key role of 1,25(OH)2D3, one of the most prevalent modifiable environmental factors in MS, in the regulation of inflammation by demonstrating its role in differentially modulating the expression of adhesion molecules involved in the migration of T cells to the CNS, depending on their activation pathways (**Figure 10**). Vitamin D decreases CD49d on both CD46- and CD28-costimulated T cells, but it only reduces CD29 in CD28-activated T cells, hence it may still allow migration of Tr1 cells through residual VLA-4 or other β1 integrins present at the surface of these cells. Of note, the analysis of the previously reported differentially expressed genes after 1,25(OH)2D3-treatment of EAE in the rat (49) shows decreased expression of *itga4* (coding for CD49d), *itgb1* (CD29) and *mcam* (CD146), suggesting, at least in part, a role of vitamin D at the transcription level. 1,25(OH)2D3 has been reported to induce a skin–homing phenotype in T cells by increasing expression of the skin-homing chemokine receptor CCR10, and decreasing expression of the gut-homing α4β7 integrin and CCR9 (41). While we detected a decrease in β7-integrin expression, we also observed a significant increase in CCR9 with addition of 1,25(OH)2D3. In the present study, we used CD4+ T cells without IL-12 stimulation, whereas the previous study (41) used predominantly CD8+ T cells that had been activated in the presence of IL-12, with minimal effects on CD4+ T cells unless these were pre-activated.

Vitamin D also strongly modulated the expression of several adhesion molecules known to control T cell migration to the CNS. Addition of 1,25(OH)2D3 *in vitro* increased expression of both CD6 and CD226, while it decreased expression of CD166 and of CD146 especially on CD46-costimulated T cells. CD146 expression is increased on T cells from Natalizumab (anti-integrin α4)-treated patients and favors Th17 migration (46, 50). The large reduction in CD146 expression observed following Vitamin D treatment would be consistent with a reduction in Th17 recruitment to the CNS. CD6 regulates T cell activation and polymorphisms have been identified in MS (45). Intriguingly, 1,25(OH)2D3 increased CD6 expression but decreased its ligand, CD166/ALCAM. It would therefore be interesting to determine the role of vitamin D on the expression of CD318, a second ligand for CD6 recently identified (51).

CD226 is expressed by Th1 and Th17 cells and its blockade ameliorates the course of EAE (52, 53). However, the role of CD226 in Tregs is controversial. Although CD226 upregulation has been associated with reduced suppressive activity (54), the level of CD226 expression in Tregs has been correlated with their increased suppressive activity, with MS Tregs expressing lower levels of CD226 (44). Furthermore, CD226 is also a marker of Tr1 (55) suggesting that it plays a role in Tr1 function. Indeed, 1,25(OH)2D3, which induces CD226 on CD46-costimulated T cells, promotes CD46-mediated Tr1 suppression (7). In EAE, ligation of CD226 has also been shown to ameliorate EAE with an increased IL-10 production (56). Furthermore, we observed that CD46 costimulation repressed TIGIT expression that was induced by both CD3 and CD3/CD28 stimulation (**Supplementary Figure S11**). TIGIT is an inhibitory co-receptor that shares CD226 ligands. Therefore, 1,25(OH)2D3-treated CD46-induced Tr1 will positively respond to CD226 ligands.

1,25(OH)2D3 also induced expression of CD99 on CD46-costimulated T cells. CD99 promotes T cell diapedesis across a human BBB *in vitro* and anti-CD99 ameliorates EAE and reduces T cell infiltration (57). Our data suggest that CD99 may however also promote migration of Tr1 cells to the CNS. Collectively, our *in vitro* data suggest that the modulation of adhesion molecules by 1,25(OH)2D3 favors migration of Tr1 cells compared with effector T cells. Indeed, 1,25(OH)2D3 increased motility of CD46-costimulated T cells in response to CXCL11, compared with CD28-activated T cells, supporting the previously reported notion that CXCL11 promotes migration of regulatory T cell subsets (58). CD46-costimulated T cells had increased VDR expression, which may explain the preferential effect of Vitamin D on these cells. Furthermore, 1,25(OH)2D3 dramatically increased miR-9 in CD46-costimulated T cells. As miR-9 targets VDR (48), we propose that the induction of miR-9 may act as a negative feedback loop to limit the effects induced by Vitamin D, which is also supported by the large induction of CYP24A1 that reduces the amount of available active vitamin D. Together, these data highlight the crosstalk between the Vitamin D and CD46 pathways, favoring the role of vitamin D in Tr1 cells. This is supported by the previous findings that vitamin D promotes IL-10-producing T cells, both *in vitro* and in humans (6, 32), and the CD46-mediated switch from Th1 to Tr1 and CD46 cleavage on activated T cells (7). This is further sustained by the negative correlation we detected between CD46 expression and circulating vitamin D levels in the cohort of patients supplemented by vitamin D.

Our *ex vivo* findings using the cohort of patients supplemented with vitamin D support an effect of vitamin D in the modulation of T cells that may be relevant to disease pathogenesis. Unsupervised t-SNE clustering analysis revealed a cluster of T cells that was decreased in the placebo group but maintained/increased after vitamin D supplementation, with lower CD46 expression but increased expression of adhesion molecules, including CD162L/PSGL1, and reduced CD146, thereby reflecting the changes observed in our *in vitro* activation conditions. This is suggestive of the activation of the CD46 pathway by vitamin D *in vivo* in these patients. However, vitamin D supplementation exerted different effects on CD226 depending on the cluster. This suggests that vitamin D will differently regulate CD226 expression depending on the T cell subsets. This may partly explain the controversial studies relating to the role of CD226 in the control of the immune response. Furthermore, the data obtained after *in vitro* activation suggest that the levels of circulating vitamin D regulate the reactivation of the T cells, modifying their phenotype in patients supplemented by vitamin D. Interestingly, the cluster 9, which is increased in the placebo group but stable with vitamin D in *ex vivo* T cells, has increased CD25 expression, which is similar to what we observed after *in vitro* reactivation of the cells. Of note, most of the patients enrolled in the vitamin D supplementation trial had almost normal vitamin D levels, and it is possible that greater changes may have been observed after supplementation with vitamin D in deficient patients. The analysis performed after *in vitro* T cell activation shows a different clustering of the cells in patients supplemented by vitamin D after CD46 costimulation, and much less so after CD28 costimulation (**Figure 8A**). These data show that vitamin D supplementation modulates the response of T cells following subsequent T cell activation (at least *in vitro*) and further support the concept that Vitamin D favors response to CD46 costimulation.

Altogether, our data provide novel key insights into the immunomodulatory role of vitamin D by exerting differential regulation of adhesion molecules on T cells, depending on their activation pathways, and suggesting that it favors the migratory ability of regulatory T cells to the CNS. This supports a beneficial role of this modifiable factor in MS.

## Data Availability Statement

The raw data supporting the conclusions of this article will be made available by the authors, without undue reservation.

## Ethics Statement

The studies involving human participants were reviewed and approved by ethical approval SR258 for MS, ethical approval AMREC 115-HV-013 for healthy donors. The patients/participants provided their written informed consent to participate in this study.

## Author Contributions

JK, JH, EM, and SV performed experiments and analyzed data. AW and JD provided MS samples. SK and TA analyzed data. SV and JD edited manuscript. AA planned the study and experiments, performed experiments, discussed data, analyzed data, and wrote manuscript. All authors contributed to the article and approved the submitted version.

## Funding

AA was funded by an RCUK fellowship and was detached from CNRS while at University of Edinburgh. These studies were supported by research grants to AA from the Multiple Sclerosis Society (MS41) and we are very grateful for a generous donation from Mrs Jessica Fincham, the Agence Nationale de la Recherche (ANR - 19 - CE14 - 0043 – 01), the Eugène Devic EDMUS Foundation against multiple sclerosis in partnership with ARSEP foundation grant to AA, ARSEP grant to AA. EM was supported by an ECTRIMS fellowship. JH held an MRC-funded PhD studentship. SV was supported by the MRC (MR/S008020/1 and MR/M023060/1).

## Conflict of Interest

The authors declare that the research was conducted in the absence of any commercial or financial relationships that could be construed as a potential conflict of interest.

## Supplementary Material

Supplementary Figure 1 Profile of adhesion molecules on activated CD4+ T cells. **(A)** Human CD4+ T cells obtained from blood were purified from PBMCs using the StemCell negative CD4+ isolation kit and were activated with anti-CD3 and anti-CD28 or anti-CD46 as indicated, in the presence or absence of 1,25(OH)2D3. **(B)** After 4 days of culture, cells were analyzed by flow cytometry for a panel of markers, as indicated. **(C)** Frozen PBMCs from MS patients supplemented with vitamin D or placebo were thawed and analyzed by flow cytometry for a panel of markers on CD4. The gating strategy is shown.

Supplementary Figure 2 1,25(OH)2D3 promotes the Th1-Tr1 switch in human CD4+ T cells. Human CD4+ T cells were activated with anti-CD3 and anti-CD28 or anti-CD46 as indicated, in the presence or absence of 1,25(OH)2D3. **(A)** Concentrations of IL-10 and IFNγ secreted were measured by ELISA. The IL-10/IFNγ ratio is shown. One way Anova with Sidak’s multiple comparisons test. **(B)** IFNγ and IL-10 secretion by CD46-costimulated T cells was assessed by secretion assay (Miltenyi).

Supplementary Figure 3 1,25(OH)2D3 differentially modulates expression of β7 integrin depending on the activation pathway. Human CD4+ T cells were left unstimulated or were activated with anti-CD3 and anti-CD28 or anti-CD46 as indicated, in the presence or absence of 1,25(OH)2D3. **(A)** Expression of integrin β7 was determined by flow cytometry (representative of 4 donors, Vitamin D=gray histograms, control=plain lines). Panel on the right shows the data obtained for the 4 donors. **(B)** Expression of CCR9 and CCR10 was quantified by qPCR (n=5 donors, mean ± SEM, one way Anova with Sidak’s multiple comparisons test).

Supplementary Figure 4 1,25(OH)2D3 modulates expression of adhesion molecules on MS CD4+ T cells. Human CD4+ T cells purified from the blood of healthy donors (HC, **Figure 1**) and patients with RRMS (see **Table 1**) were left unstimulated (US) or were activated with anti-CD3 and anti-CD28 or anti-CD46 as indicated, in the presence or absence of 1,25(OH)2D3. After 4 days, expression of CD49d, CD11a, CD29, CD99, CD146, CD166, CD6 and CD226 was assessed by flow cytometry (MS: mean ± SEM, n=17, Friedman test with Dunn’s multiple comparison test and Kruskall-Wallis test).

Supplementary Figure 5 The modulation of adhesion molecules by 1,25(OH)2D3 is not affected by treatments. Human CD4+ T cells purified from the blood of patients with RRMS (see **Table 1**) were left unstimulated (US) or were activated with anti-CD3 and anti-CD28 or anti-CD46 as indicated, in the presence or absence of 1,25(OH)2D3. After 4 days, expression of CD49d, CD29, CD6, CD146, CD166, and CD226 was assessed by flow cytometry (UT=untreated; DMF=dimethyl fumarate; IFNβ=beta-interferon).

Supplementary Figure 6 Effect of 1,25(OH)2D3 on CXCR3 expression. CXCR3 expression on unstimulated, CD3, CD3/CD46 or CD3/CD28-co-activated T cells from healthy donors in the presence or absence of 1,25(OH)2D3 (n=25, mean ± SEM).

Supplementary Figure 7 t-SNE clustering representation of the effects of Vitamin D supplementation on T cells in patients with MS. **(A)** t-SNE representation and **(B)** intensity of staining of CD46, CD25, CD162, CD45RA, CD127, CD146, CD6, Foxp3 and CD226 in CD3+CD4+ T cell clusters of PMBCs from patients with MS supplemented by vitamin D or placebo (n=5 and n=4, respectively).

Supplementary Figure 8 1,25(OH)2D3 induces PSGL-1 on CD46-costimulated T cells. Human CD4+ T cells purified from the blood of healthy donors were coactivated with anti-CD3 and anti-CD28 or anti-CD46 as indicated, in the presence or absence of 1,25(OH)2D3. After 4 days, expression of PSGL-1/CD162 was assessed by flow cytometry (mean ± SEM, n=8, Friedman test with Dunn’s multiple comparison test).

Supplementary Figure 9 Vitamin D supplementation does not specifically modulate the proportion of Tregs in patients with MS. **(A)** Identification of the Treg clusters (Foxp3^hi^CD25^hi^CD127^lo^) by PhenoGraph analysis and their phenotype (5 patients supplemented with vitamin D and 4 placebo (all IFNβ treated). **(B)** Percentage and count of these two Treg clusters in the vitamin D and placebo groups before and after treatment.

Supplementary Figure 10 Vitamin D supplementation modulates the phenotype of T cells in patients with MS after *in vitro* activation. Percentage of the 19 CD3+CD4+ T cell clusters identified by PhenoGraph in the vitamin D and placebo groups (n=3 and n=4, respectively) before and after treatment after CD28 or CD46 costimulation of PMBCs. Clusters increased (green boxes) or decreased (blue boxes) after vitamin D supplementation are indicated.

Supplementary Figure 11 CD46-costimulation represses TIGIT expression on activated T cells. Human CD4+ T cells purified from the blood of healthy donors were coactivated with anti-CD3 and anti-CD28 or anti-CD46 as indicated, in the presence or absence of 1,25(OH)2D3. After 4 days, expression of TIGIT was assessed by flow cytometry (mean ± SEM, n=5, Friedman test with Dunn’s multiple comparison test).
