# Supplementary material for: Vitamin D/CD46 Crosstalk in Human T Cells in Multiple Sclerosis
Source: Front Immunol. 2020 Nov 24;11:598727. doi: 10.3389/fimmu.2020.598727 (PMC7732696; doi:10.3389/fimmu.2020.598727)
Supplement: Supplementary Figure 1 — Profile of adhesion molecules on activated CD4+ T cells. (A) Human CD4+ T cells obtained from blood were purified from PBMCs using the StemCell negative CD4+ isolation kit and were activated with anti-CD3 and anti-CD28 or anti-CD46 as indicated, in the presence or absence of 1,25(OH)2D3. (B) After 4 days of culture, cells were analyzed by flow cytometry for a panel of markers, as indicated. (C) Frozen PBMCs from MS patients supplemented with vitamin D or placebo were thawed and analyzed by flow cytometry for a panel of markers on CD4. The gating strategy is shown. [file DataSheet_1.pdf]

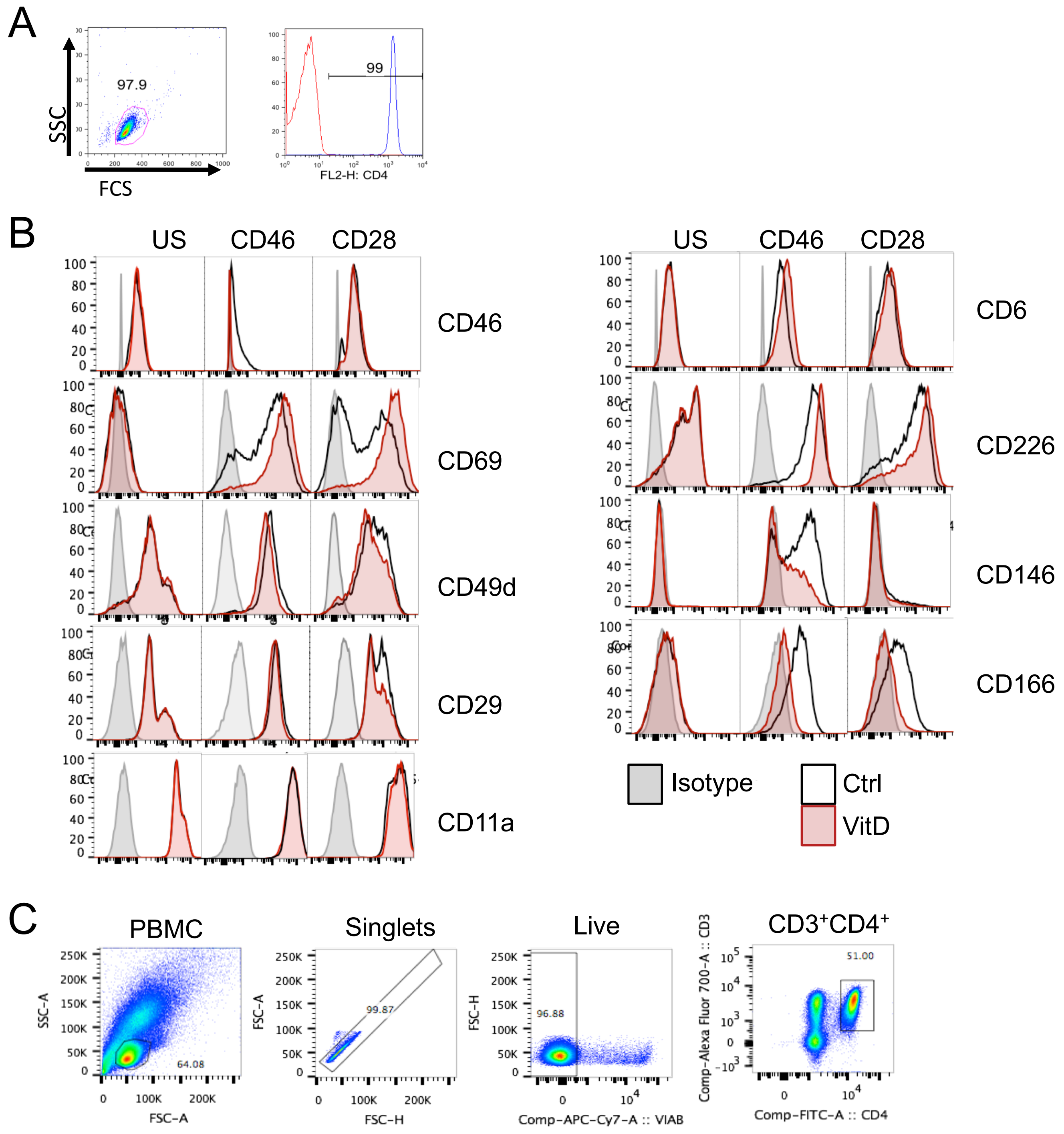
A
97.9
SSC
FCS
99
FL2-H: CD4
B
US
CD46
CD28
CD46
CD69
CD49d
CD29
CD11a
CD6
CD226
CD146
CD166
Isotype
Ctrl
VitD
C
PBMC
64.08
SSC-A
FSC-A
Singlets
99.87
FSC-A
FSC-H
Live
96.88
FSC-H
Comp-APC-Cy7-A :: VIAB
CD3+CD4+
51.00
Comp-Alexa Fluor 700-A :: CD3
Comp-FITC-A :: CD4

Supp Fig S1

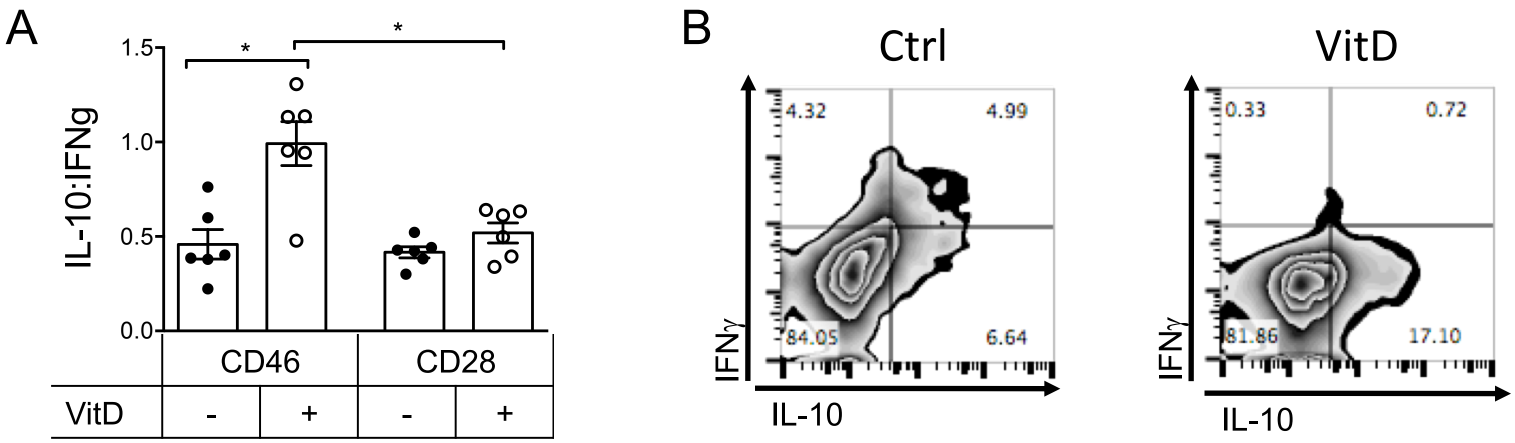
A
B
1.5
1.0
0.5
0.0
IL-10:IFNg
*
*
CD46
CD28
VitD
-
+
-
+
Ctrl
VitD
4.32
4.99
84.05
6.64
0.33
0.72
81.86
17.10
IFNγ
IL-10
IFNγ
IL-10

Supp Fig S2

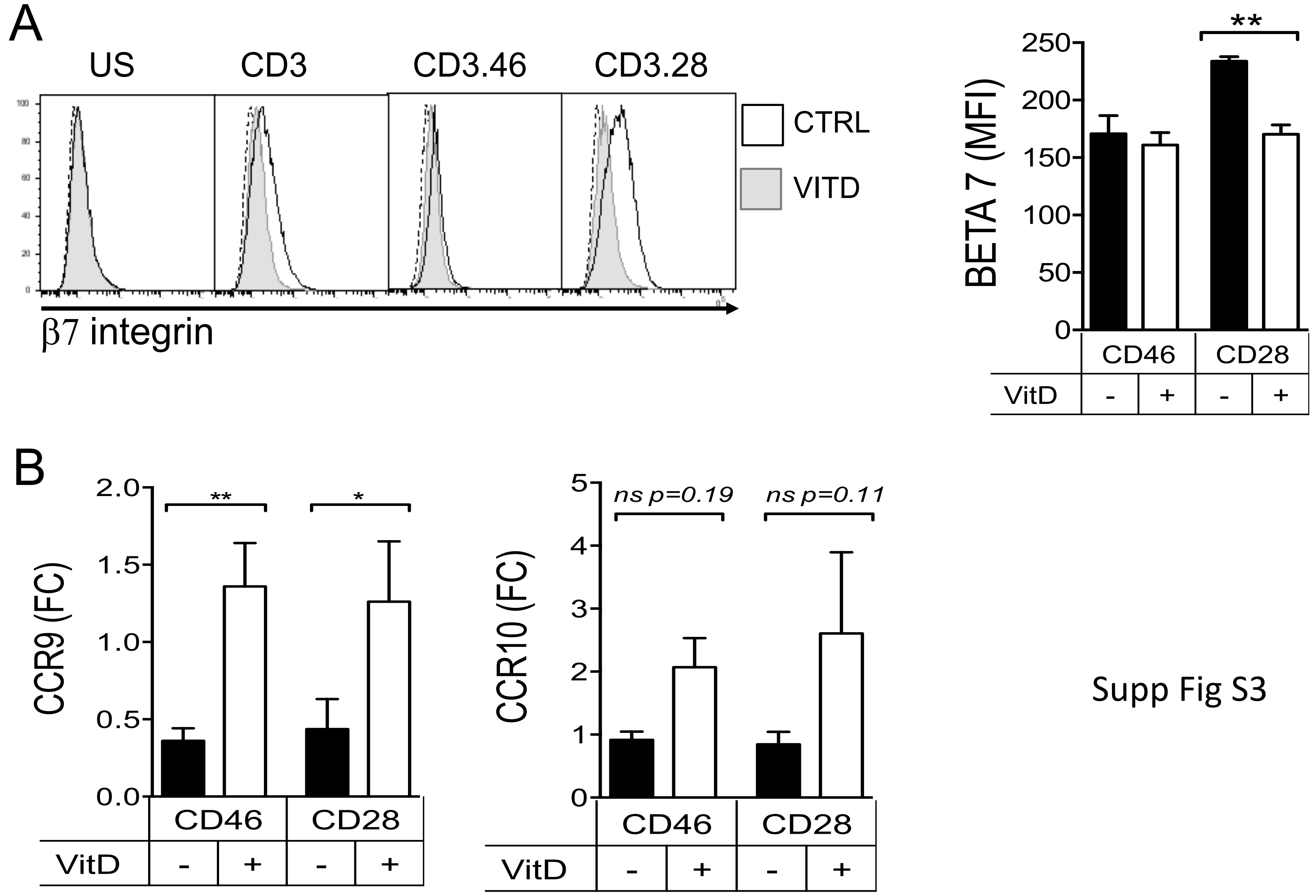
A
US
CD3
CD3.46
CD3.28
CTRL
VITD
β7 integrin
BETA 7 (MFI)
**
CD46
CD28
VitD
-
+
B
CCR9 (FC)
CCR10 (FC)
**
*
ns p=0.19
ns p=0.11
CD46
CD28
VitD
-
+

Supp Fig S3

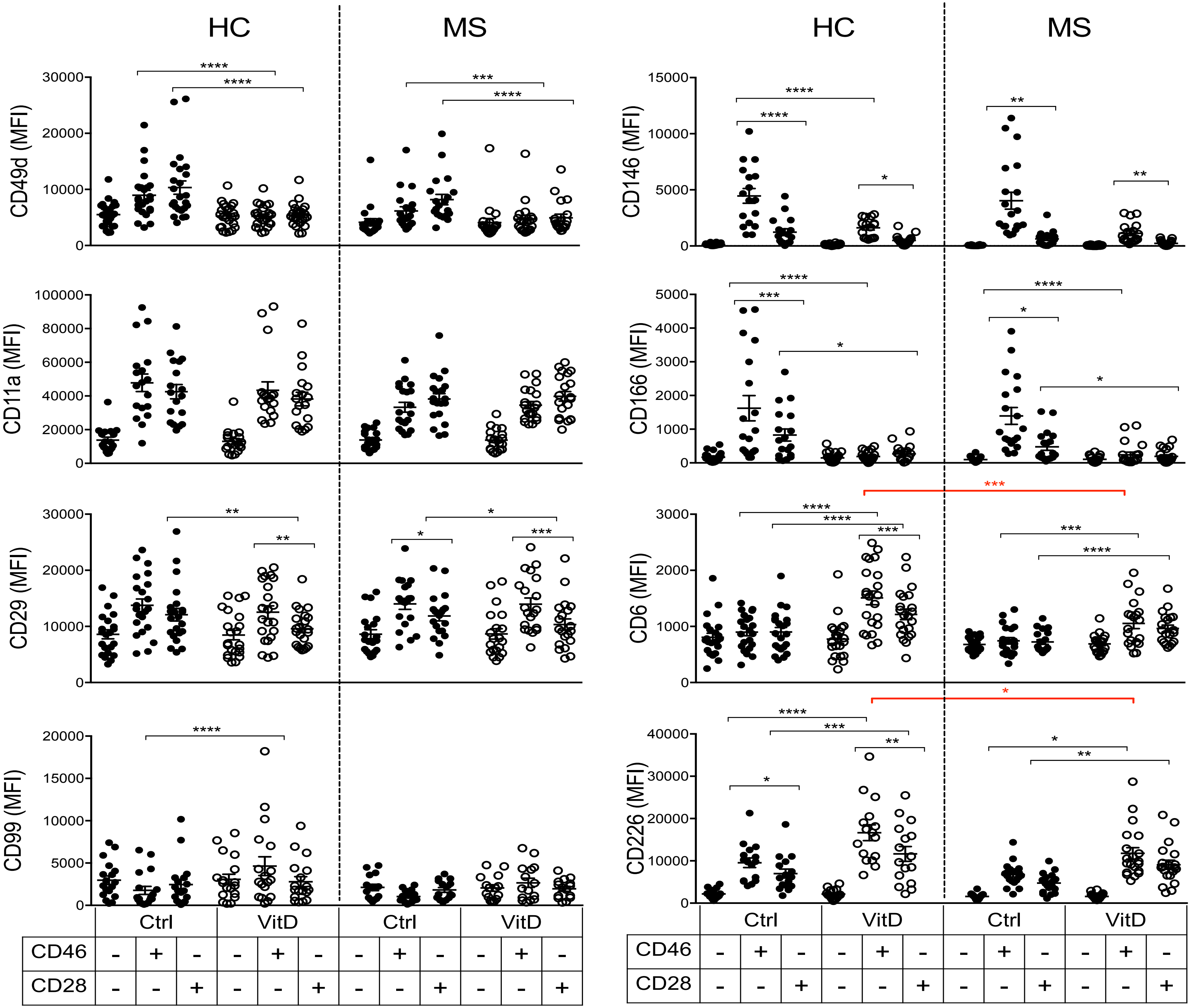

HC
MS
CD49d (MFI)
CD11a (MFI)
CD29 (MFI)
CD99 (MFI)
CD146 (MFI)
CD166 (MFI)
CD6 (MFI)
CD226 (MFI)
Ctrl
VitD
CD46
CD28

Supp Fig S4

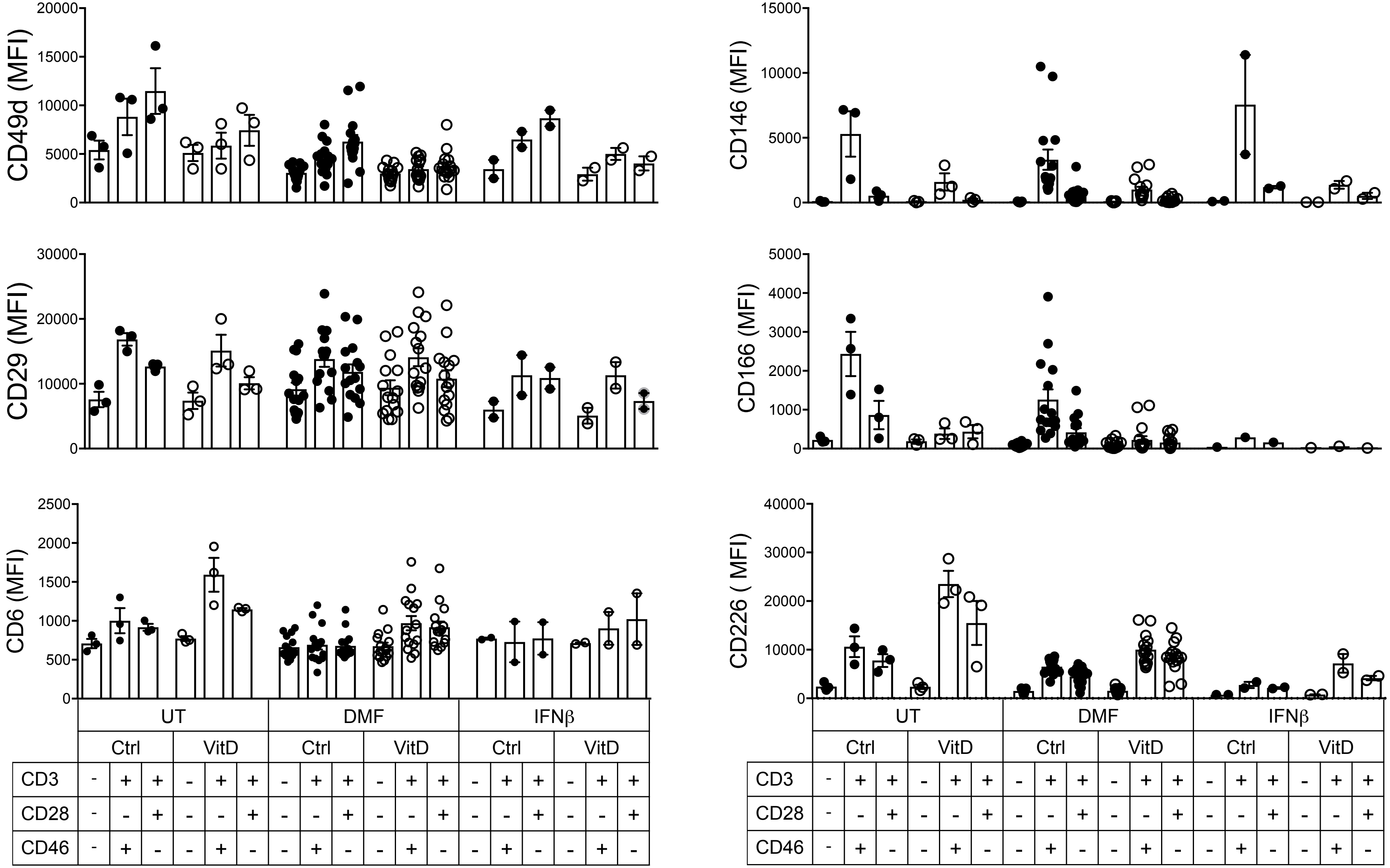

CD49d (MFI)
CD146 (MFI)
CD29 (MFI)
CD166 (MFI)
CD6 (MFI)
CD226 (MFI)
UT
DMF
IFNβ
Ctrl
VitD
CD3
CD28
CD46

Supp Fig S5

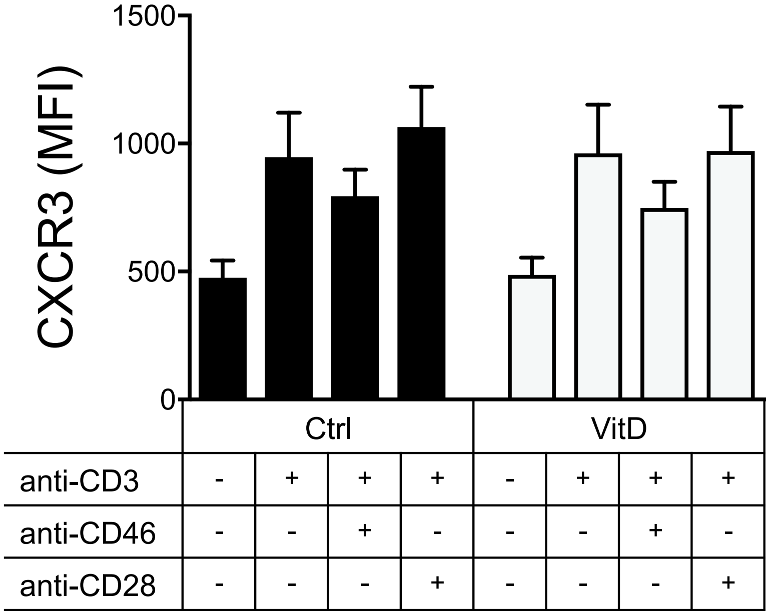
CXCR3 (MFI)
1500
1000
500
0
Ctrl
VitD
anti-CD3
anti-CD46
anti-CD28
- + + + - + + +
- - + - - - + -
- - - + - - - +

Supp Fig S6

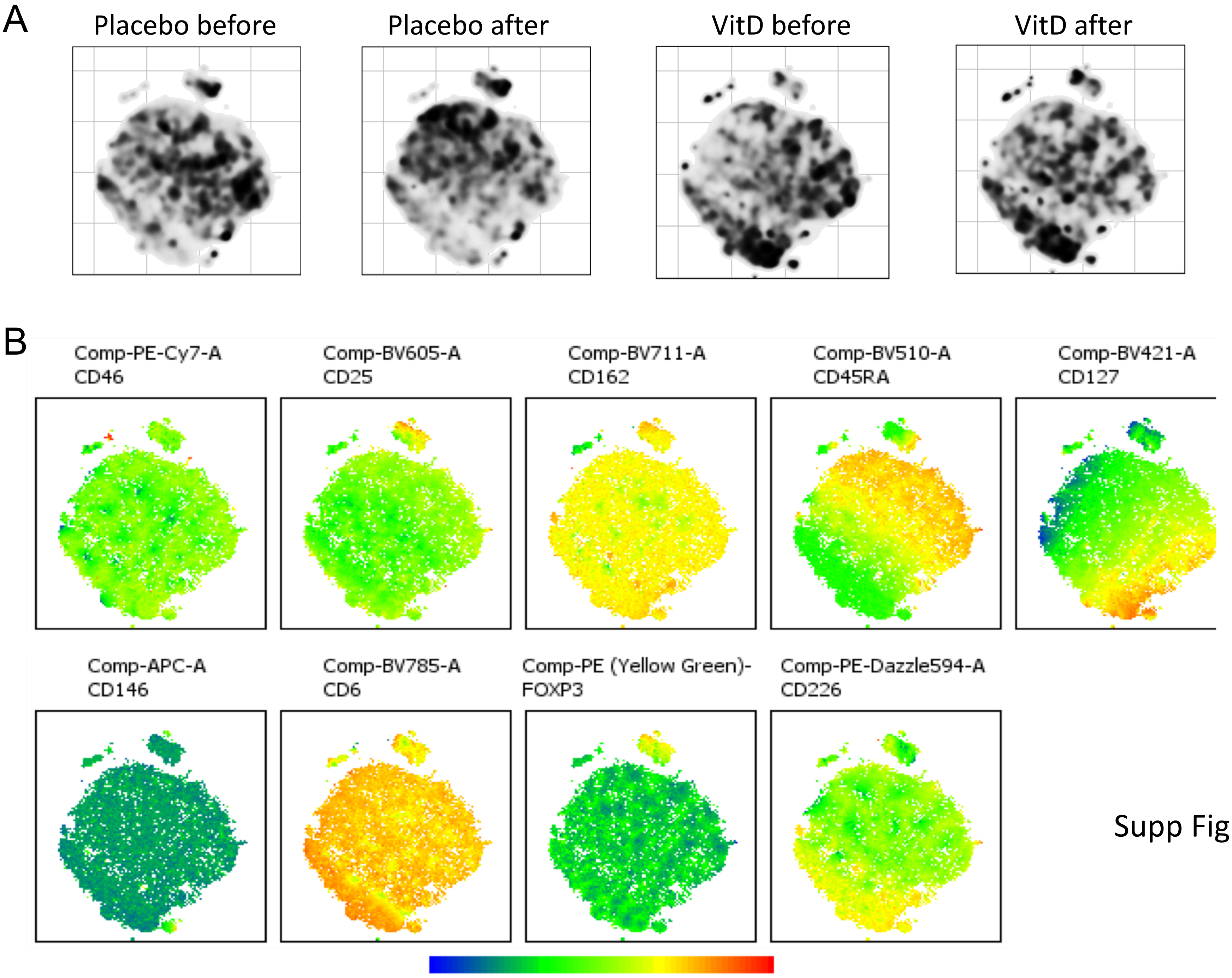
A
Placebo before
Placebo after
VitD before
VitD after
B
Comp-PE-Cy7-A
CD46
Comp-BV605-A
CD25
Comp-BV711-A
CD162
Comp-BV510-A
CD45RA
Comp-BV421-A
CD127
Comp-APC-A
CD146
Comp-BV785-A
CD6
Comp-PE (Yellow Green)-
FOXP3
Comp-PE-Dazzle594-A
CD226

Supp Fig S7

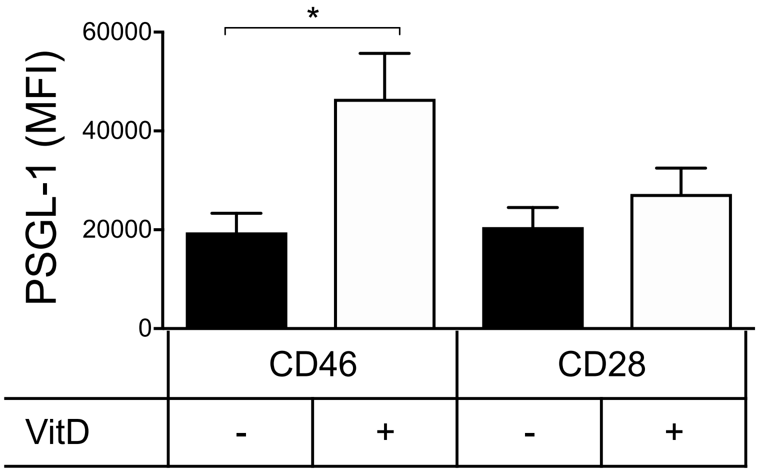

Supp Fig S8

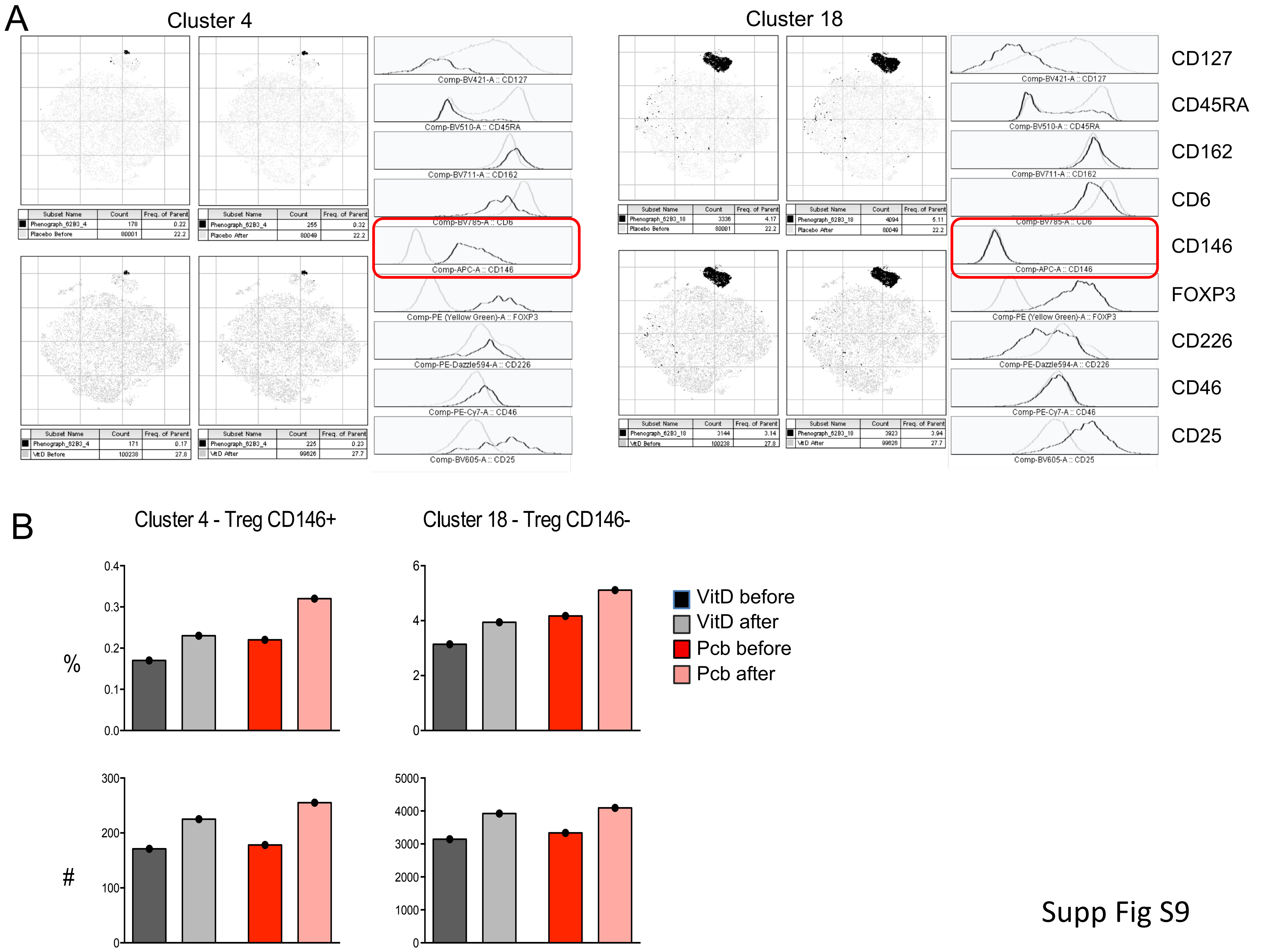
A
Cluster 4
Cluster 18
CD127
CD45RA
CD162
CD6
CD146
FOXP3
CD226
CD46
CD25
Comp-BV421-A :: CD127
Comp-BV510-A :: CD45RA
Comp-BV711-A :: CD162
Comp-BV785-A :: CD6
Comp-APC-A :: CD146
Comp-PE (Yellow Green)-A :: FOXP3
Comp-PE-Dazzle594-A :: CD226
Comp-PE-Cy7-A :: CD46
Comp-BV605-A :: CD25
Subset Name Count Freq. of Parent
Phenograph_62B3_4 178 0.22
Placebo Before 80001 22.2
Phenograph_62B3_4 255 0.32
Placebo After 80049 22.2
Phenograph_62B3_4 171 0.17
VitD Before 100238 27.8
Phenograph_62B3_4 225 0.23
VitD After 99626 27.7
Phenograph_62B3_18 3336 4.17
Placebo Before 80001 22.2
Phenograph_62B3_18 4094 5.11
Placebo After 80049 22.2
Phenograph_62B3_18 3144 3.14
VitD Before 100238 27.8
Phenograph_62B3_18 3923 3.94
VitD After 99626 27.7
B
Cluster 4 - Treg CD146+
Cluster 18 - Treg CD146-
%
#
VitD before
VitD after
Pcb before
Pcb after

Supp Fig S9

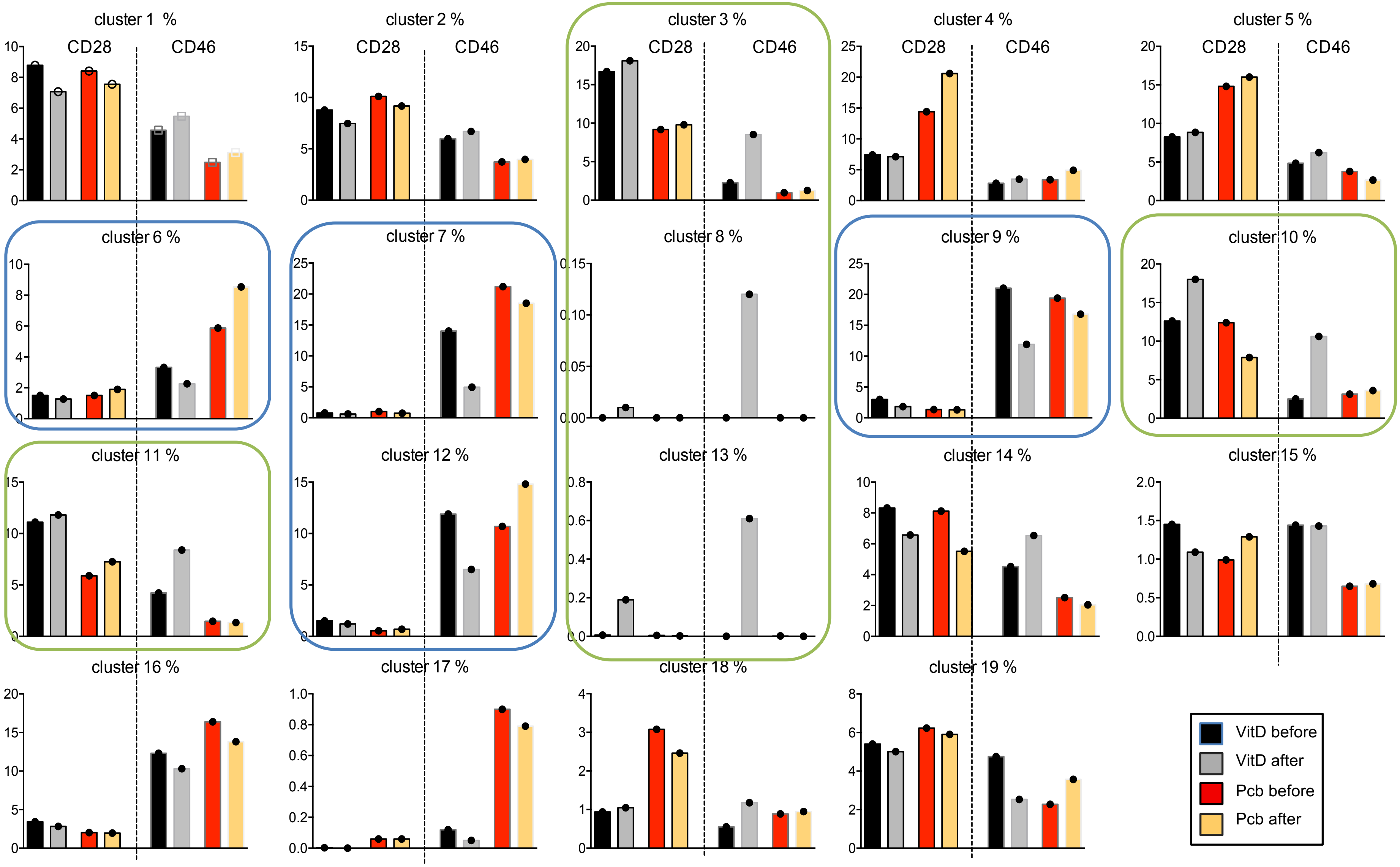
cluster 1 %
CD28
CD46
cluster 2 %
cluster 3 %
cluster 4 %
cluster 5 %
cluster 6 %
cluster 7 %
cluster 8 %
cluster 9 %
cluster 10 %
cluster 11 %
cluster 12 %
cluster 13 %
cluster 14 %
cluster 15 %
cluster 16 %
cluster 17 %
cluster 18 %
cluster 19 %
VitD before
VitD after
Pcb before
Pcb after

Supp Fig S10

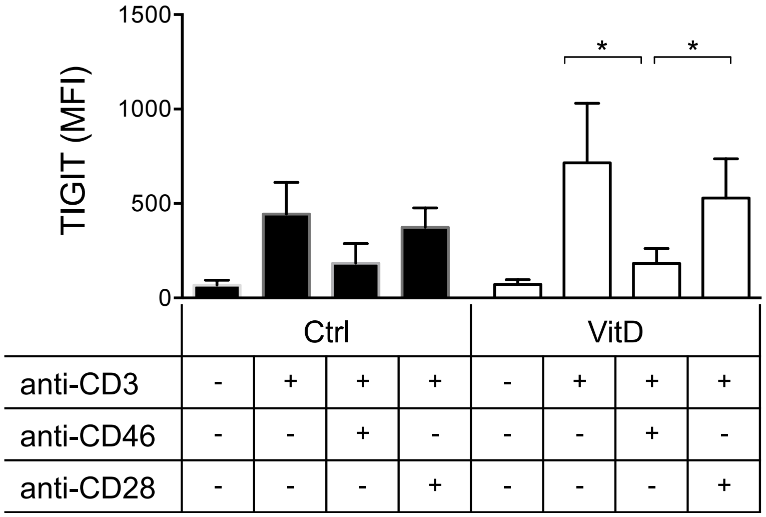
1500
1000
500
0
TIGIT (MFI)
*
*
Ctrl
VitD
anti-CD3
anti-CD46
anti-CD28
- + + + - + + +
- - + - - - + -
- - - + - - - +

Supp Fig S11
